# Socio-Demographic, Behavioral and Psychological Factors Associated with High BMI among Adults in a Southeast Asian Multi-Ethnic Society: A Structural Equation Model

**DOI:** 10.3390/nu15081826

**Published:** 2023-04-10

**Authors:** Han Shi Jocelyn Chew, Shaun Seh Ern Loong, Su Lin Lim, Wai San Wilson Tam, Nicholas W. S. Chew, Yip Han Chin, Ariana M. Chao, Georgios K. Dimitriadis, Yujia Gao, Bok Yan Jimmy So, Asim Shabbir

**Affiliations:** 1 Alice Lee Centre for Nursing Studies, Yong Loo Lin School of Medicine, National University of Singapore, Singapore 117597, Singapore; 2 Yong Loo Lin School of Medicine, National University of Singapore, Singapore 117597, Singapore; 3 Dietetics Department, National University Hospital, Singapore 119074, Singapore; 4 Department of Cardiology, National University Heart Centre, Singapore 119074, Singapore; 5 Department of Biobehavioral Health Sciences, School of Nursing, University of Pennsylvania, Philadelphia, PA 19104-4217, USA; 6 Department of Endocrinology ASO/EASO COM, King’s College Hospital NHS Foundation Trust, Denmark Hill, London SE5 9RS, UK; 7 Obesity, Type 2 Diabetes and Immunometabolism Research Group, Department of Diabetes, Faculty of Cardiovascular Medicine & Sciences, School of Life Course Sciences, King’s College London, London WC2R 2LS, UK; 8 Division of Hepatobiliary & Pancreatic Surgery, Department of Surgery, National University Hospital, Singapore 119074, Singapore; 9 Division of General Surgery (Upper Gastrointestinal Surgery), Department of Surgery, National University Hospital, Singapore 119074, Singapore

**Keywords:** obesity, overweight, sociodemographic, body weight determinants, weight loss, weight management, self-regulation, lifestyle habits, waist circumference

## Abstract

While various influencing factors of overweight and obesity have been identified, the underlying mechanism remains unclear. We examined the relationships among sociodemographic, behavioral, and psychological factors on anthropometry in a multi-ethnic population with overweight and obesity. Participants (*N* = 251) were recruited from January to October 2022. Mean age and self-reported BMI were 31.7 ± 10.1 years and 29.2 ± 7.2 kg/m^2^. Participants were mostly female (52.4%) and overweight (58.2%). Multivariate multiple regression was performed using maximum likelihood estimation. Body mass index was associated with waist circumference, age, sex, race, marital status, education level, residential region, overeating habit, immediate thinking, self-regulation, and physical activity, but not anxiety, depression, or the intention to change eating habits. Final model indicated good fit: χ^2^ (30, *N* = 250) = 33.5, *p* = 0.32, CFI = 0.993, TLI = 0.988, RMSEA = 0.022, and SRMR = 0.041. Direct effects were found between BMI and overeating (β = 0.10, *p* = 0.004), race (β = −0.82, *p* < 0.001), marital status (β = −0.42, *p* = 0.001), and education level (β = −0.28, *p* = 0.019). Crisps (68.8%), cake (66.8%) and chocolate (65.6%) were identified as the most tempting foods. Immediate thinking indirectly increased overeating habits through poor self-regulation, although sociodemographic characteristics better predicted anthropometry than psycho-behavioral constructs.

## 1. Introduction

The global prevalence of overweight and obesity has more than doubled since 1980 [1], affecting approximately 2.5 billion (52%) adults in 2016 [2]. In 2022, the World Health Organization released an updated report, which stated that 60% of the adults in Europe were living with overweight or obesity [3]. Overweight and obesity, hereinafter referred to as having a high BMI, is a complex biopsychosocial condition that centers around the concept of energy balance [4]. According to the Foresight obesity system map, the problem of obesity involves 108 unique influencing factors and more than 300 lines of association [4]. Concepts and theories have been used to explain the biological, social, and behavioral determinants of obesity [5], one of which is the hypothesis that chronic emotional conditions activate the hypothalamic–pituitary–adrenal (HPA) stress response system that alters metabolism, contributing to weight gain [6]. According to the life course theory, people who grow up in lower socioeconomical statuses (SES) or have experienced inequalities, are more susceptible to having a high BMI due to the greater access to cheaper yet unhealthy food [7,8]. Moreover, some people are more likely to over-compensate for their early years’ low access to healthy food by overeating when they are more financially stable in their later years [9]. Similarly, the fundamental cause theory posits that people of higher SES tend to have better health outcomes due to the greater access to the disease prevention resources needed [10]. According to the health lifestyle theory, structural factors, such as age, gender, ethnicity, and living conditions, interact to form the foundation for habit formation, lifestyle and health outcomes [11]. These theories highlight the importance of the structural variables that influence weight status though psychosocial behavioral factors. However, the interplay among these factors remains unclear. 

Even in developed countries, such as Singapore, food insecurity exists. With a median salary of SGD10 099 [12], approximately 10% of local households experience food insecurity at least once a year. This is despite Singapore being ranked as the most food-secure country in the world in 2019 (28th in 2022). Considerable efforts have been put forth to address issues on food security through policy and built environment restructuring. For example, residents from lower income households are given food vouchers or rations through an assistance scheme set up by the Ministry of Social and Family Development to increase the access to nutritious and culturally appropriate foods [13]. However, in a 2020 report, only 22% of these households with food insecurity receive support [14]. Town planning efforts have also been improved in an attempt to increase the number of affordable food options near government-subsidized housing, e.g., in neighborhood coffee shops [15]. However, the effectiveness of such programs, especially the newer initiatives, remains unclear. 

In order to better understand the interplay between the influencing factors of having a high BMI, various statistical models have been tested. Most of the existing models have considered variables including demographic factors (e.g., age, marital status, and SES (e.g., education, employment, and income level)) [16,17,18,19], behavioral factors (e.g., diet, sleep and physical activity) [16,17,18,19], and health status (e.g., the presence of medical conditions) [17,18]. However, most of these studies were conducted on western populations, which may not be generalizable to a Southeast Asian population. Differences could arise from the strong influence of ethnicity on weight-related factors, such as genetic predisposition, [20] food choices, [21] and body image preference [22]. Moreover, such studies were often not modeled based on bio-psycho-behavioral theories, which could provide a deeper insight on the problem. Adults with high BMI often intend to improve eating behaviors but fail to do so due to the momentary succumbing to food temptations, a decisional conflict known as temporal discounting [23,24,25]. Temporal discounting is a phenomenon in which people prefer a smaller sooner reward rather than a larger later reward, a construct that has also been shown to mediate the relationship among anxiety, depression, and unhealthy health-related habits [24,25]. According to the Temporal Self-Regulation Theory (TST), such a behavioral misalignment could be due to the presence of a strong habit and weak self-regulation capacity [26]. In addition to psycho-behavioral constructs, studies have shown that habitual overeating and eating in response to negative emotional states are associated with changes in the neurocircuits comprising the basal ganglia, the amygdala, and the prefrontal cortex, which are brain centers for self-regulation [27]. These findings highlight the importance of the concept of self-regulation in influencing body weight. 

Given the complex interplay among bio-psycho-behavioral factors, we examined the relationship among several factors in a multi-ethnic, high-income Southeast Asian population with overweight and obesity. Based on the existing literature, we hypothesized that BMI and waist circumference would be influenced by an overeating habit, which would be directly influenced by immediate thinking and indirectly through self-regulation. Additionally, we hypothesized that immediate thinking and an overeating habit would be directly influenced by anxiety and depression. Lastly, we hypothesized that race, marital status, highest attained education level, and physical activity would have direct effects on BMI and waist circumference.

## 2. Materials and Methods

Results presented in this paper are based on the baseline data obtained from a study on the effectiveness of an artificial intelligence-assisted weight loss app on food consumption. The study was approved by the National Healthcare Group (NHG) Domain Specific Review Board (DSRB) Ethics Review Board (ref: 2020/01439) and registered with ClinicalTrials.gov (ref. NCT04833803). This study is reported according to the STrengthening the Reporting of OBservational studies in Epidemiology (STROBE) checklist for cross-sectional studies (Table S1). 

### 2.1. Setting and Participants

Participants were recruited from January 2022 to October 2022 from the public and a local tertiary hospital’s weight management clinic through social media and physical recruitment, respectively. Written informed consent was obtained before recruitment. Participants were included if they: (1) were ≥21 years old; (2) had a BMI ≥ 23 kg/m^2^, (3) were able to comprehend the English language; (4) were using a smartphone; and (5) were able to provide informed consent. Participants were excluded if they were enrolled in a commercial weight loss program. A sample size of 247 was calculated to be sufficient in representing the local population with overweight (28.8%), with a margin of error of 5.7% at 80% power.

### 2.2. Data Collection and Variables Assessed

Data were collected through Qualtrics, an online survey platform.

#### 2.2.1. Sociodemographic and Anthropometric Profile

Data on age, sex, marital status, race, religion, highest education level, employment, and per capita household income were collected through a survey. BMI and waist circumference were self-reported during the recruitment survey and app onboarding. Participants were instructed on how to measure their height and weight, which were reported to the study team members in order to calculate their respective BMIs. The Asian cut-off scores were used for both BMI and waist circumference (Table 1) [28].

#### 2.2.2. Intention to Change Eating Behaviors

Intention was measured using the question, “I intend to change my eating behaviors to lose weight?” Responses were measured on a 7-point Likert scale (1 = Strongly Disagree; 2 = Disagree; 3 = Somewhat Disagree; 4 = Neutral; 5 = Somewhat Agree; 6 = Agree; 7 = Strongly Agree) [29].

#### 2.2.3. Self-Regulation of Eating Behavior

Self-regulation of eating behavior was measured on a five-point scale (1 = Never; 2 = Rarely; 3 = Sometimes; 4 = Frequently; 5 = Always), using the five-item Self-regulation of Eating Behavior Questionnaire (SREBQ) (α = 0.75) [30]. It encompasses constructs such as “self-monitoring, appraising progress, … and the capacity to control behavior, thoughts and attention”. An example of an item is, “I’m good at resisting tempting food”. A mean score (three reverse-coded items) of <2.8 represents low self-regulation, 2.8–3.6 represents moderate, and >3.6 represents high self-regulation. This questionnaire also includes a section to identify, from a list, the food items that participants find most tempting. The validity of SREBQ was previously established with a strong positive correlation among general measures of self-regulation, motivation, and behavioral automaticity [30]. It has also been shown to be negatively correlated with food responsiveness and emotional overeating [30].

#### 2.2.4. Consideration of Future Consequences

Consideration of future consequences was measured on a 7-point scale using the 6-item Consideration of future consequences scale (CFCS-6) [31]. CFC refers to the extent to which one considers the future consequences of current behaviors, and has been established as a cognitive motivational construct that influences behaviors related to energy balance [32]. It comprises two subscales (immediate [reverse-coded] and future) on which a higher average sum represents more future-oriented thinking. Both subscales demonstrated good internal reliability (α_immediate_ = 0.821; α_future_ = 0.775) [32]. 

#### 2.2.5. Overeating Habit

Habit strength of overeating and snacking was measured on a 7-point scale using the 12-item Self-Report Habit Index (SRHI) [33]. It consists of three factors, namely automaticity; behavior frequency; and self-identity. SRHI has been commonly used to measure behaviors related to energy balance (e.g., physical activity and unhealthy diet) [34]. SRHI has been shown to be reliable upon a 1-week test–retest (α = 0.90; *p* < 0.001), and valid when correlated with the response-frequency measure of habit (r = 0.58, *p* < 0.001) [33].

#### 2.2.6. Physical Activity

Physical activity over the past 7 days was measured using the 7-item International Physical Activity Questionnaire Short-Form (IPAQ-SF). The IPAQ-SF assessed duration, frequency, exertion level, and amount (vigorous, moderate, and walking) of physical activity. Ref. [35] Scores were transformed to Metabolic Equivalent Task (MET) by multiplying day and minute scores according to the guidelines (walking-, moderate-, and vigorous-intensity activity correspond to 3.3 METs, 4.0 METs, and 8.0 METs, respectively). Scores were summed and classified into low (<600 METmin/wk), moderate (≥600 METmin/wk), and high (≥3000 MET min/wk) physical activity. Its validity has been well established with accelerometry [36].

#### 2.2.7. Depression and Anxiety

Depression and anxiety were measured using the two-item Patient Health Questionnaire-2 (PHQ-2) [37] and two-item Generalized Anxiety Disorder-2 (GAD-2) [38], respectively. A four-point scale (1 = not at all; 2 = several days; 3 = more than half the days; 4 = nearly every day) was used. Sum scores of ≥3 for each scale represented possible cases of depression and anxiety, respectively.

### 2.3. Data Analysis

R (lavaan), AMOS version 28, and IBM SPSS were used for data management, visualization, and analysis. Descriptive statistics were reported in mean ± standard deviation (SD) for continuous variables, and frequency (%) for categorical variables. Missing data on all questionnaires (*n* = 3) were managed by imputating means, except for IPAQ-SF scores, which were managed by listwise deletion, as recommended by Ref. [39]. Categorical variables with more than two levels (binary) were replaced with dummy variables. Pearson’s correlations were used to examine the correlations among the variables of interest, and ordinary least square regression was used to examine the relationship among the variables in the SEM model. BMI and waist circumference estimates were converted to Z-scores in order to establish a normal distribution. 

Structural equation modeling (SEM) was used to examine the complex direct and indirect relationships among study variables. A path analysis (multivariate multiple regression) was performed using the maximum likelihood estimation (MLE). Model fit was assessed using the chi-square statistic (χ^2^), comparative fit index (CFI), Tucker–Lewis Index (TLI), and Root Mean Square Error of Approximation (RMSEA) and standardized root mean square residual (SRMR). Good model fit was indicated by χ^2^ test *p*-value > 0.05, CFI ≥ 0.95, TLI ≥ 0.95, RMSEA ≤ 0.08, and SRMR < 0.05. Refs. [40,41] Direct and indirect effects were examined by bootstrapping at 10,000 iterations. Post hoc subgroup analyses were conducted using one-way ANOVA on the mean differences in self-regulation, overeating habit, consideration of future consequences, physical activity, anxiety, depression, and BMI and waist circumference. Post hoc tests were performed using the Bonferroni correction, and Games-Howell was used if equal variance was not assumed. 

## 3. Results

Of the 251 participants enrolled in the study, one participant was later found to have a BMI < 23 kg/m^2^ and was removed from the analyses. Of the 250 included participants, the mean age was 31.7 ± 10.1 years, and mean BMI was 29.2 ± 7.2 kg/m^2^. Most of the participants were single (70.4%), Chinese (75.2%), Buddhists (30.7%), employed full-time (75.6%), non-smoking (95.2%), had a university education (63.7%) and a per capita household income of SGD 5001–10,000 (34.0%). The participants’ characteristics are detailed in Table 1.

Scores on intention to change eating behaviors to lose weight (5.7 ± 1.2) suggested that most participants somewhat agreed that they intend to change their eating behaviors in order to lose weight. Most of the participants had a moderate level of self-regulation of eating behaviors (n = 150, 59.8%) and a low level of physical activity (n = 88, 35.2%). The top three foods that were identified as the most tempting were crisps (68.8%), cake (66.8%), and chocolate (65.6%). Scores on each variable are detailed in Table 2.

BMI was correlated with waist circumference, age, behavioral frequency (overeating), CFCi, CFCt, SREBQ, and total MET-min/week (Tables S2 and S3: zero-order correlation tables). The correlates for waist circumference were similar to those of weight, except that sex was a significant correlate, while SREBQ and total MET-min/week were not (Table S2). Interestingly, intention to change eating behaviors was directly correlated with CFCi and behavior frequency (overeating) but not BMI or self-regulation. Two regression models were tested. In the first model, only psycho-behavioral constructs (i.e., anxiety, depression, immediate thinking, self-regulation, and overeating habit) were entered, which explained 6.6% of the adjusted variance in BMI and that only immediate thinking was a significant predictor of BMI (B = 0.012, *p* = 0.02). In the second model, additional sociodemographic characteristics (i.e., age, race, marital status, education level, and residential region) were entered, which explained 26.9% of the adjusted variance in BMI, and only marital status (single) (B = −0.43, *p* = 0.04), race (Chinese) (B = −0.74, *p* < 0.001), education level (university) (B = −0.28, *p* = 0.02), and residence (west) (B = 0.28, *p* = 0.02) were significant predictors of BMI.

**Figure 1:**
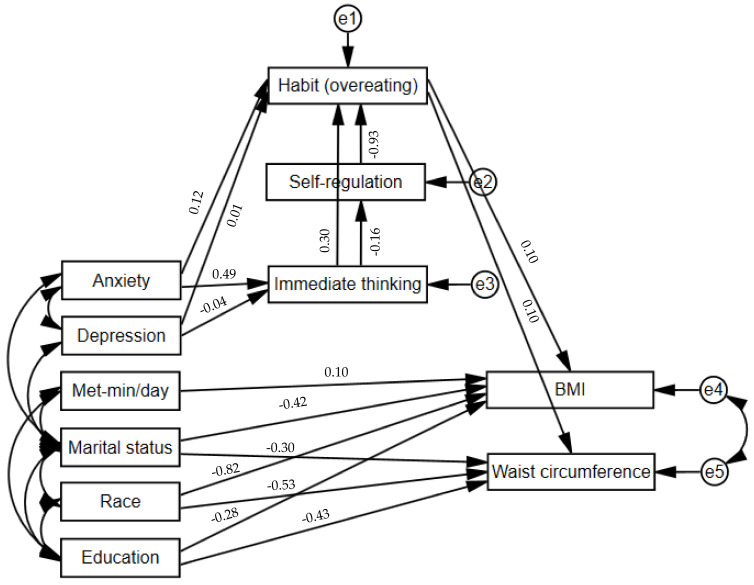
Hypothesized conceptual model that explains the relationships among the examined variables. Note: Met-min/day = Metabolic Equivalent Task–minutes/day; BMI = body mass index; e1–e5 represent individual error terms for each endogenous/dependent variable. Double-arrowed lines represent covariances. Path coefficients of each relationship are shown on each path.

The final model indicated a good fit: χ^2^ (30, N = 250) = 33.5, *p* = 0.30, CFI = 0.993, TLI = 0.988, RMSEA = 0.022, and SRMR = 0.041 (Figure 1). As hypothesized, habit (overeating) had a direct effect on both BMI (β = 0.10, *p* = 0.004) and waist circumference (β = 0.10, *p* = 0.005). We observed a direct effect (β = 0.30, *p* < 0.001) between immediate thinking and habit (overeating) and an indirect effect through self-regulation (β = 0.15, *p* < 0.001). Depression did not have a significant effect on immediate thinking and habit (overeating), and anxiety had a direct effect on immediate thinking (β = 0.49, *p* = 0.003) but not on habit (overeating) (β = 0.12, *p* = 0.41). Direct effects were found between BMI and race (Chinese) (β = −0.82, *p* < 0.001), marital status (single) (β = −0.42, *p* = 0.001), and highest attained education level (β = −0.28, *p* = 0.019). Direct effects were also found between waist circumference and race (Chinese) (β = −0.53, *p* < 0.001), marital status (single) (β = −0.30, *p* = 0.02), and highest attained education level (university) (β = −0.43, *p* < 0.001). This model explained 26.9% of the variance in BMI and 16.8% of the variance in waist circumference.

### Post Hoc Subgroup Analysis

Subgroup analyses demonstrated significant differences in BMI among ethnicities (F (3246) = 30.9, *p* ≤ 0.001), marital statuses (F (2247) = 12.1, *p* ≤ 0.001), and education levels (F (3246) = 8.92, *p* ≤ 0.001). Individuals of the Malay race were found to have a significantly higher BMI than those of the Chinese race (mean difference (95% confidence interval; CI) = −9.87 (−14.79 to −4.94), *p* < 0.001). People who were single (−4.05 (−6.40 to −1.70), *p* ≤ 0.001) and divorced (−11.77 (−21.50 to 6.40), *p* = 0.012) were found to have a significantly lower BMI than those who were married. People with a university education were also found to have a lower BMI than people with other education levels. There were no statistically significant differences in BMI and waist circumference for the other variables included in Figure 1.

## 4. Discussion

Significant associations were found among sociodemographic factors (race, education, and marital status), physical factors (MET), psychological factors (depression and anxiety), behavioral factors (overeating), and anthropometric measurements (BMI and waist circumference). The conceptual model was tested to be a good fit to the sample data, which could potentially serve as a guiding framework for the design and development of weight management interventions in a similar population. Interestingly, sociodemographic characteristics played a greater role in influencing BMI and, while around a quarter and a fifth of our sample had high risks of anxiety and depression, these risks were not significant predictors of BMI. 

Coinciding with previous studies, we found that married individuals were more likely to have a higher BMI and waist circumference. This coincides with previous studies, in which married men and women were significantly more likely to be overweight and have abdominal obesity than unmarried adults [42]. While the exact reasons underlying the association between obesity and marital status are not fully understood, several hypotheses have been described. The social obligation hypothesis states that, in a marriage, couples potentially eat more regular meals, along with richer and denser foods [42,43]. Another possible hypothesis is the marriage market hypothesis, which suggests that married couples are less concerned about attracting others in the marriage market [42,44]. This suggests that future weight management efforts may want to target married dyads in order to enhance the efficiency of such programs.

Additionally, we found that individuals of the Malay race were more likely to have higher BMI and waist circumference. This coincides with existing local studies that ethnic Malays and Indians were more likely to live with obesity, as compared to ethnic Chinese individuals [45,46]. This could be related to various entrenched cultural and genetic factors, for which large scale genome-wide studies have found associations between population-specific characteristics and levels of obesity [47,48,49,50,51]. Cultural and lifestyle factors include having a lower level of physical activity and higher consumption of unhealthy food [52,53,54,55]. 

Our findings also correspond with existing studies, which report that having a higher level of education is significantly associated with lower BMI and waist circumference [42,56,57]. In the US, one study based on National Health and Nutrition Examination Survey (NHANES) data reported that the prevalence of obesity was lower among adults with a college education, regardless of sex [58]. This could be due to the self-reinforcing effect, whereby having a higher educational qualification increases one’s access to updated information, healthier food alternatives, and resources to maintain a healthy lifestyle [59,60,61,62]. However, this observation could be highly subjective to the population in question, as the study also reported that there were no differences in obesity prevalence among non-Hispanic Asians, regardless of their education level and household income [58]. This suggests the potential influence of other obesogenic factors such as genetics, culture, and lifestyle. Moreover, amenities surrounding the built environment could play a role in influencing the mobility patterns of a certain ethnically populated region, thereby influencing the residents’ level of physical activity and weight status [63]. 

Interestingly, we found that having an intention to change eating behaviors was correlated with immediate thinking and the behavior frequency of overeating, but not with BMI nor waist circumference. This suggests that having the intention to improve eating behaviors may not be sufficient to result in significant anthropometric changes when accounting for an actual behavior change, as an intention to change does not always translate to an actual behavior [64]. More research is needed to examine the temporal decision-making process and strategies that can better translate an intention into a behavior [65]. For instance, we found that self-regulation mediated the relationship between immediate thinking and the frequency of overeating, indicating that strengthening self-regulation skills could dampen the effects of dietary triggers and temptations, thus promoting successful dietary inhibition and weight loss. 

Consistent with other studies [66,67,68], having a higher tendency for immediate thinking was associated with lower self-regulation, which is associated with a higher likelihood of overeating. This could be explained by the negative affect regulation model for overeating, whereby individuals with a reduced self-regulation capacity are more likely to rely on eating as a coping mechanism for stressors in life [69]. This is consistent with our finding that anxiety has a direct effect on immediate thinking, although its association with overeating did not reach significance. This could be related to the need to act immediately, as triggered by the harm-avoidance mechanisms when one feels anxious, often in response to a threatening situation [70]. It is, however, interesting that anxiety and depression were not associated with overeating, in contrast to earlier studies which found otherwise [71]. This could be attributed to factors such as an insufficient sample size and, thus, power to detect such associations in the context of mental health conditions. The insignificant finding could have also been due to the difficulty in obtaining accurate mental health assessment in a conservative Asian society, where topics of weight status and mental health remain taboo [72,73]. Therefore, results from this segment should be carefully weighed against the backdrop of mental health stigma, and clinicians should still be aware of the link between mental health conditions and weight [74]. 

### Strengths and Limitations

This study is the first to characterize sociodemographic factors, time perspective, self-regulation, and eating behaviors in relation to obesity and overweight in a Southeast Asian sample. The study paves the way for specific interventions to target identified modifiable risk factors in this and similar population groups. However, this study also has its limitations. Firstly, the recruitment of participants using social media could have limited the generalizability of the study findings to this population. While this is a SEM of a south-east Asian country, the extent to which the findings can be generalized to other countries in the region awaits replications. Secondly, BMI and waist circumference were self-reported, which may have introduced recall biases and inconsistencies to the study findings. Secondly, the use of questionnaires such as the PHQ-2 and GAD-2 might be insufficient for the accurate confirmation of depression and anxiety, which, while acceptable in sensitivity and specificity, could potentially be improved using PHQ-9 and GAD-7 as a secondary test if the individual tests positive in the present questionnaire. These additional steps would improve the reliability of identifying anxiety and depression in cohorts Thirdly, this study was cross-sectional; thus, the directionality of relationships cannot be determined. 

## 5. Conclusions

We found that sociodemographic characteristics were more predictive than the examined psycho-behavioral constructs in influencing BMI and waist circumference. Future studies could examine the mediators of this phenomenon in order to develop more effective and personalized weight management interventions. This study provides a robust and holistic understanding of the complex sociodemographic–psychological–behavioral–physical interplay in this landscape of obesity and its determinants in a high-income country like Singapore. Our findings could inform future weight management interventions, such as ethnicity-specific initiatives aimed at modifying ethnicity-specific obesity or overweight risk factors Our findings could also be used to improve evidenced-based guidelines for the prevention and management of obesity and overweight. 

## 6. Study Importance

Several studies have investigated the relationship between various sociodemographic and/or psycho-behavioral risk factors and overweight and obesity. However, few studies have identified these relationships concurrently in Southeast Asian populations.

Sociodemographic characteristics were more predictive than the examined psycho-behavioral constructs in influencing BMI and waist circumference. Future studies could examine the mediators of this phenomenon in order to develop more effective and personalized weight management interventions.

## Figures and Tables

**Table 1:** Sociodemographic characteristics of the 250 study participants.

Characteristics of Participants	Mean ± SD/Frequency (%)
* Age, years	31.7 ± 10.1 (28.0; 24.0–27.0)
Young adults (21–35 years old)	179 (71.6)
Middle aged adults (36–64 years old)	71 (28.4)
Sex	
Males	119 (47.6)
Females	131 (52.4)
Marital status	
Single	176 (70.4)
Married	71 (28.4)
Divorced	3 (1.2)
Race	
Chinese	188 (75.2)
Indian	29 (11.6)
Malay	26 (10.4)
Others	7 (2.8)
Religion	
Buddhism	77 (30.7)
Christianity	60 (24.0)
Hinduism	18 (7.2)
Islam	35 (14.0)
Freethinker	46 (18.4)
Others	14 (5.6)
Highest educational level	
Primary school	2 (0.8)
Secondary school	11 (4.4)
Pre-university	77 (30.8)
University	160 (63.7)
Per capita household income (SGD/month)	
<1000	23 (9.2)
1000–3000	53 (21.2)
3001–5000	60 (24.0)
5001–10,000	85 (34.0)
>10,000	29 (11.6)
Residential region	
Central	44 (17.6)
East	20 (8.0)
North	23 (9.2)
Northeast	37 (14.8)
West	81 (32.4)
Smoking	
No	238 (95.2)
Yes	12 (4.8)
Employment	
Part-time	52 (20.8)
Full-time	189 (75.6)
Retired	9 (3.6)
* Body mass index, kg/m^2^,	29.2 ± 7.2 (26.4; 24.3–31.3)
Overweight (23.0–24.9 kg/m^2^; moderate risk)	84 (33.6)
Obese I (25.0–29.9 kg/m^2^; high risk)	95 (38.0)
Obese II (≥30.0 kg/m^2^; very high risk)	71 (28.4)
BMI Z-score	0.003 ± 1
* Waist circumference, cm	93.4 ± 18.9 (89; 81.3–99.0)
High (male ≥ 90 cm; female ≥ 80 cm)	171 (68.4)
Waist Circumference Z-score	0.001 ± 1

Note: SD = standard deviation; SGD = Singapore dollars; * Medians; interquartile range (IQR) are presented in parentheses.

**Table 2:** Mean scores of the 250 study participants on each psychological and physical variable.

Characteristics of Participants	Mean ± SD/Frequency (%)
Intention to change eating behaviors	5.7 ± 1.2
SRHI (overeating)	4.3 ± 1.5
Behavioral frequency (overeating)	4.4 ± 1.6
Automaticity (overeating)	4.3 ± 1.6
Self-identify (overeating)	4.0 ± 1.6
SRHI (snacking)	4.2 ± 1.4
Behavioral frequency (snacking)	4.5 ± 1.6
Automaticity (snacking)	4.0 ± 1.6
Self-identify (snacking)	4.1 ± 1.5
CFCS-6 total	4.4 ± 1.1
CFCS-6 immediate	4.3 ± 1.6
CFCS-6 future	5.0 ± 1.1
SREBQ	2.9 ± 0.5
Low	90 (35.9)
Moderate	149 (59.6)
High	11 (4.4)
GAD-2	1.79 ± 1.60
Potentially at risk (≥3)	61 (24.4)
PHQ-2	1.54 ± 1.59
Potentially at risk (≥3)	52 (20.8)
IPAQ-SF, MET-min/week	2184.4 ± 2557.4
Low	88 (35.2)
Moderate	65 (26.0)
High	85 (34.0)
Identifies the following foods as tempting:	
Chocolate	164 (65.6)
Crisps	172 (68.8)
Cake	167 (66.8)
Ice cream	151 (60.4)
Fried foods	149 (59.6)
Chips	118 (47.2)
Bread/toast	109 (43.6)
Pastries	102 (40.8)
Pizza	100 (40.0)
Biscuits	60 (24.0)
Fizzy drinks	59 (23.6)
Sweets	46 (18.6)
Others	41 (16.4)
Popcorn	37 (14.8)
Nil	8 (3.2)

Note: SRHI = Self-Report Habit Index; CFCS-6 = Consideration of Future Consequences Scale-6; SREBQ = Self-Regulation of Eating Behavior Questionnaire; GAD-2 = Generalized Anxiety Disorder two-item questionnaire; PHQ-2 = Patient Health Questionnaire two-item questionnaire; IPAQ = International Physical Activity Questionnaire; MET-min/week = Metabolic Equivalent Task—minutes/week.

## Data Availability

Data are available upon request from the corresponding author.

## Supplementary Materials

The following supporting information can be downloaded at: https://www.mdpi.com/article/10.3390/nu15081826/s1, Table S1. STROBE Statement—checklist of items that should be included in reports of observational studies. Table S2. Correlations (Pearson correlation coefficient [r]) between BMI, waist circumference, age, sex, race (Chinese), marital status (single), education level (university), residential region (west). Table S3. Correlations (Pearson correlation coefficient [r]) between BMI, waist circumference, intention to change eating behaviour, behavioural frequency (snacking), automaticity (snacking), self-identity (snacking), behavioural frequency (overeating), automaticity (overeating), self-identity (overeating), immediate, future, and total consideration of future consequences, self-regulation of eating behaviour, anxiety, depression, and physical activity.

## Clinical trial registration

ClinicalTrials.gov (ref. NCT04833803), registered on 6 April 2021.

## Abbreviations

BMI	body mass index
HPA	hypothalamic–pituitary–adrenal
SES	socioeconomical status
TST	Temporal Self-Regulation Theory
NHG	National Healthcare Group
DSRB	Domain Specific Review Board
STROBE	STrengthening the Reporting of OBservational studies in Epidemiology
SREBQ	Self-regulation of Eating Behavior Questionnaire
CFCS	Consideration of future consequences scale
SRHI	Self-Report Habit Index
IPAQ-SF	International Physical Activity Questionnaire Short-Form
MET	Metabolic Equivalent Task
PHQ	Patient Health Questionnaire
GAD	Generalized Anxiety Disorder
SEM	Structural equation modeling
MLE	maximum likelihood estimation
CFI	comparative fit index
TLI	Tucker–Lewis Index
RMSEA	Root Mean Square Error of Approximation
CI	confidence interval
