# Supplementary material for: Socio-Demographic, Behavioral and Psychological Factors Associated with High BMI among Adults in a Southeast Asian Multi-Ethnic Society: A Structural Equation Model
Source: Nutrients. 2023 Apr 10;15(8):1826. doi: 10.3390/nu15081826 (PMC10144460; doi:10.3390/nu15081826)
Supplement: Supplementary file 1 [file nutrients-15-01826-s001.zip › nutrients-2240140-supplementary.pdf]

Table S1. STROBE Statement—checklist of items that should be included in reports of observational studies

	Item No.	Recommendation	Page No.	Relevant text from manuscript
**Title and abstract**	1	(*a*) Indicate the study's design with a commonly used term in the title or the abstract	1	
		(*b*) Provide in the abstract an informative and balanced summary of what was done and what was found	3-4	
**Introduction**				
Background/rationale	2	Explain the scientific background and rationale for the investigation being reported	5-7	
Objectives	3	State specific objectives, including any prespecified hypotheses	7	
**Methods**				
Study design	4	Present key elements of study design early in the paper	7-8	
Setting	5	Describe the setting, locations, and relevant dates, including periods of recruitment, exposure, follow-up, and data collection	8	
Participants	6	(*a*) *Cohort study*—Give the eligibility criteria, and the sources and methods of selection of participants. Describe methods of follow-up<br>*Case-control study*—Give the eligibility criteria, and the sources and methods of case ascertainment and control selection. Give the rationale for the choice of cases and controls<br>*Cross-sectional study*—Give the eligibility criteria, and the sources and methods of selection of participants	8	
		(*b*) *Cohort study*—For matched studies, give matching criteria and number of exposed and unexposed<br>*Case-control study*—For matched studies, give matching criteria and the number of controls per case	NA	
Variables	7	Clearly define all outcomes, exposures, predictors, potential confounders, and effect modifiers. Give diagnostic criteria, if applicable	8-10	

Data sources/ measurement	8*	For each variable of interest, give sources of data and details of methods of assessment (measurement). Describe comparability of assessment methods if there is more than one group	8-10
Bias	9	Describe any efforts to address potential sources of bias	8-10
Study size	10	Explain how the study size was arrived at	8
Quantitative variables	11	Explain how quantitative variables were handled in the analyses. If applicable, describe which groupings were chosen and why	10-11
Statistical methods	12	(*a*) Describe all statistical methods, including those used to control for confounding	10-11
		(*b*) Describe any methods used to examine subgroups and interactions	10-11
		(*c*) Explain how missing data were addressed	10-11
		(*d*) *Cohort study*—If applicable, explain how loss to follow-up was addressed<br>*Case-control study*—If applicable, explain how matching of cases and controls was addressed<br>*Cross-sectional study*—If applicable, describe analytical methods taking account of sampling strategy	NA
		(*e*) Describe any sensitivity analyses	NA
**Results**			
Participants	13*	(a) Report numbers of individuals at each stage of study—eg numbers potentially eligible, examined for eligibility, confirmed eligible, included in the study, completing follow-up, and analysed	11
		(b) Give reasons for non-participation at each stage	NA
		(c) Consider use of a flow diagram	NA
Descriptive data	14*	(a) Give characteristics of study participants (eg demographic, clinical, social) and information on exposures and potential confounders	Tables 1 and 2
		(b) Indicate number of participants with missing data for each variable of interest	11
		(c) *Cohort study*—Summarise follow-up time (eg, average and total amount)	

Outcome data	15*	*Cohort study*—Report numbers of outcome events or summary measures over time	
		Case-control study—Report numbers in each exposure category, or summary measures of exposure	
		Cross-sectional study—Report numbers of outcome events or summary measures	11-13
Main results	16	(*a*) Give unadjusted estimates and, if applicable, confounder-adjusted estimates and their precision (eg, 95% confidence interval). Make clear which confounders were adjusted for and why they were included	10-11
		(*b*) Report category boundaries when continuous variables were categorized	NA
		(*c*) If relevant, consider translating estimates of relative risk into absolute risk for a meaningful time period	NA

Other analyses	17	Report other analyses done—eg analyses of subgroups and interactions, and sensitivity analyses	13
**Discussion**			
Key results	18	Summarise key results with reference to study objectives	13-17
Limitations	19	Discuss limitations of the study, taking into account sources of potential bias or imprecision. Discuss both direction and magnitude of any potential bias	16-17
Interpretation	20	Give a cautious overall interpretation of results considering objectives, limitations, multiplicity of analyses, results from similar studies, and other relevant evidence	16-17
Generalisability	21	Discuss the generalisability (external validity) of the study results	16-17
**Other information**			
Funding	22	Give the source of funding and the role of the funders for the present study and, if applicable, for the original study on which the present article is based	NA

*Give information separately for cases and controls in case-control studies and, if applicable, for exposed and unexposed groups in cohort and cross-sectional studies.

Note: An Explanation and Elaboration article discusses each checklist item and gives methodological background and published examples of transparent reporting. The STROBE checklist is best used in conjunction with this article (freely available on the Web sites of PLoS Medicine at http://www.plosmedicine.org/, Annals of Internal Medicine at http://www.annals.org/, and Epidemiology at http://www.epidem.com/). Information on the STROBE Initiative is available at www.strobe-statement.org.

Table S2. Correlations (Pearson correlation coefficient [*r*]) between BMI, waist circumference, age, sex, race (Chinese), marital status (single), education level (university), residential region (west).

	BMI	Waist circumference	Age	Sex	Race	Marital status	Education
Waist circumference	.620**						
Age	.139*	.161*					
Sex	0.046	-.157*	-0.048				
Race	-.400**	-.277**	0.012	-0.047			
Marital status	-.276**	-.186**	-.541**	0.049	.155*		
Education	-.236**	-.260**	-.165**	-0.047	.167**	.134*	
Residential region	.190**	0.122	0.123	-0.093	-0.097	-.169**	-0.051

Note: *$p<0.05$, **$p<0.01$

Table S3. Correlations (Pearson correlation coefficient [*r*]) between BMI, waist circumference, intention to change eating behaviour, behavioural frequency (snacking), automaticity (snacking), self-identity (snacking), behavioural frequency (overeating), automaticity (overeating), self-identity (overeating), immediate, future, and total consideration of future consequences, self-regulation of eating behaviour, anxiety, depression, and physical activity.

		BMI	1	2	3	4	5	6	7	8	9	10	11	12	13	14
1	WC	.620**														
2	Intention	0.097	-0.046													
3	Behavioural frequency (overeating)	.133*	.137*	.141*												
4	Automaticity (overeating)	0.032	0.023	.131*	.859**											
5	Self-identity (overeating)	0.082	0.089	.128*	.878**	.860**										
6	Behavioural frequency (snacking)	0.012	0.088	.164**	.347**	.288**	.346**									
7	Automaticity (snacking)	-0.014	0.083	.181**	.325**	.357**	.386**	.763**								
8	Self-identity (snacking)	-0.111	0.034	.141*	.342**	.314**	.444**	.844**	.841**							
9	CFCS-6 immediate	.200**	.191**	.163**	.454**	.441**	.464**	.428**	.459**	.435**						
10	CFCS-6 future	-0.079	-0.115	.125*	-0.089	-0.067	-0.053	-.223**	-.161*	-.175**	-.375**					
11	CFCS-6 total	-.180**	-.191**	-0.053	-.364**	-.343**	-.353**	-.411**	-.403**	-.392**	-.890**	.757**				
12	SREBQ	-.127*	-0.096	0.052	-.446**	-.419**	-.412**	-.390**	-.380**	-.377**	-.490**	.337**	.512**			
13	GAD-2	0.066	0.056	0.111	.157*	0.089	.141*	.172**	.160*	.184**	.210**	-0.082	-.189**	-0.091		
14	PHQ-2	.137*	0.105	0.101	0.105	0.079	0.086	0.123	.127*	0.091	.147*	-0.111	-.158*	-0.111	.660**	
15	IPAQ-SF	-.191**	-0.089	-0.025	0.022	-0.005	-0.026	0.045	-0.059	0.007	-0.011	0.057	0.036	0.052	0.044	0.039

Note: WC=waist circumference; CFCS-6 = Consideration of Future Consequences Scale-6; SREBQ = Self-Regulation of Eating Behaviour Questionnaire; GAD-2 = Generalized Anxiety Disorder 2-items; PHQ-2 = Patient Health Questionnaire 2-items; *$p<0.05$, **$p<0.01$
